# Clinical associations of mucin 1 in human lung cancer and precancerous lesions

**DOI:** 10.18632/oncotarget.26278

**Published:** 2018-11-02

**Authors:** Andreas Saltos, Farah Khalil, Michelle Smith, Jiannong Li, Michael Schell, Scott J. Antonia, Jhanelle E. Gray

**Affiliations:** ^1^ Department of Thoracic Oncology, H. Lee Moffitt Cancer Center and Research Institute, Tampa, Florida, USA; ^2^ Department of Anatomic Pathology, H. Lee Moffitt Cancer Center and Research Institute, Tampa, Florida, USA; ^3^ Department of Biostatistics/Bioinformatics, H. Lee Moffitt Cancer Center and Research Institute, Tampa, Florida, USA

**Keywords:** lung cancer, tumor pathology, glycoprotein, MUC1, non-small cell lung cancer

## Abstract

Mucin 1 (MUC1) is a cell membrane glycoprotein overexpressed in non-small cell lung cancer (NSCLC) and has been implicated in carcinogenesis of premalignant lung lesions. Thus, MUC1 has been a target of interest for vaccine strategies for lung cancer treatment and prevention. Here, we assessed MUC1 expression by immunohistochemistry using tumor samples from patients with biopsy-proven NSCLC. Levels of expression in areas of dysplasia, metaplasia, adenocarcinoma *in situ*, and carcinoma within the same tissue sample were characterized independently on a scale of 0–3 for paired comparison. We also assessed clinical data for correlations with MUC1 expression. Our analysis included 16 samples from patients with squamous lesions and 19 from patients with adenocarcinoma lesions. Among squamous lesions, MUC1 expression score was significantly increased in dysplastic compared with metaplastic areas (mean difference = 0.83, 95% confidence interval [CI], 0.21-infinity; *P* = 0.021). MUC1 expression was also increased among areas of squamous cell carcinoma versus dysplastic areas (mean difference = 0.44, 95% CI, −0.006-infinity; *P* = 0.052). In the adenocarcinoma lesions, MUC1 expression was increased in adenocarcinoma versus adenocarcinoma *in situ*, although not significantly (mean difference = 0.20, 95% CI, −0.055-infinity; *P* = 0.094). The increase in MUC1 expression with the progression of premalignant lung lesions to invasive carcinoma in patients with NSCLC supports MUC1 as a possible therapeutic target for the prevention and treatment of lung cancer.

## INTRODUCTION

Lung cancer is the most common cause of cancer mortality in the United States for both men and women [1]. Approximately 80% of lung cancer patients are diagnosed with non-small cell lung cancer (NSCLC), of which adenocarcinoma and squamous cell carcinoma are the most common subtypes [2]. Despite recent advances in the understanding of the tumor biology and mutations in lung cancer, outcomes remain poor, particularly for patients with advanced-stage disease or disease that persists after multiple lines of therapy.

Mucin 1 (MUC1) is a cell membrane glycoprotein overexpressed in many human cancers [3–5], including NSCLC [6–9]. The mucin family of glycoproteins plays a role in the formation of mucus and contributes to its viscosity and the lubrication of mucosal-epithelial surfaces [10]. MUC1 consists of 2 subunits: the C-terminal subunit MUC1-C (which contains a small cytoplasmic domain and transmembrane domain) and the N-terminal subunit MUC1-N (consisting of a large extracellular domain of tandem repeats that is heavily glycosylated) [11–13]. MUC1 is anchored as a membrane-bound protein in the apical surface of a variety of human epithelial tissues, but the MUC1-N subunit is also released into the circulation in a soluble form [3, 4]. Recent studies have demonstrated that the membrane-bound MUC1-C also interacts with a variety of signaling pathways associated with cancer, including EGFR and NF-kB [11–13].

MUC1 is normally expressed in a polarized fashion on the apical cell membrane, and studies have demonstrated more depolarized expression in cancerous cells, which may assist in metastasis due to inhibition of cell-matrix and cell-cell adhesion [5, 14]. Increased cell surface expression of MUC1 has also been shown to interfere with T-cell interactions, allowing tumor cells to evade cellular immune responses [5, 10]. There is not only a lack of polarization but also altered hypoglycosylation of the MUC1 glycoprotein in lung cancers, which leads to uncovered protein epitopes and greater immunogenicity [3, 4, 6–8]. In addition, the soluble form of MUC1 has been demonstrated to be toxic to T-cells, in part by inhibiting interleukin 2 production and thus lymphocyte proliferation [15].

A number of *in vivo* studies support the role of MUC1 in carcinogenesis. A previously published study found that gastric cancer cell lines that were transfected with MUC1 demonstrated increased invasiveness [16]. Increased tissue expression of MUC1 has been implicated in the malignant progression of type II pneumocytes in animal (hamster) models [10], as well as for mammary carcinoma in mouse models [17].

Regarding the role of MUC1 in NSCLC, several clinical studies have demonstrated a negative prognostic association of tumor MUC1 overexpression in NSCLC [14, 18–20]. MUC1 has been shown to be overexpressed or aberrantly expressed in both adenocarcinoma and squamous carcinoma NSCLC, as well as in premalignant lesions, including squamous metaplasia and squamous dysplasia [18, 21]. However, the role of MUC1 expression in the transformation of premalignant lung lesions into invasive carcinoma is less well defined. For this study, we hypothesized that the degree of MUC1 expression increases during the development of human lung cancer, thus serving as an important target of cancerous and precancerous lesions.

## RESULTS

### Patient characteristics

Of 38 assessed tumor samples from patients with biopsy-proven NSCLC, 16 patients with squamous and 19 patients with adenocarcinoma lesions had tumor samples that were satisfactory for analyses. Baseline characteristics for both groups of patients are summarized in Table 1. Most patients had stage I or stage II tumors.

**Table 1:** Baseline patient characteristics

	Number of patients	
	Squamous (*n* = 16)	Adenocarcinoma (*n* = 19)
Median age (range), years	67 (33–82)	69 (52–84)
<65 years	5	5
≥65 years	11	14
Sex		
Male	9	7
Female	7	12
Median pack-years (range)	50 (15–80)	36 (0–105)
0 pack-years	0	6
1–29 pack-years	2	3
≥30 pack-years	14	10
Stage		
I	10	13
II	4	4
III	1	2
IV	1	0
Mutations		
None or unknown	16	13
KRAS	0	3
EGFR	0	3

Mutation information was available for 10 of 19 patients with adenocarcinoma, although none had metastatic disease at the time of tumor sampling. No mutation information was available for patients with squamous tumors. EGFR, epidermal growth factor receptor; KRAS, Kirsten rat sarcoma viral oncogene homolog.

### MUC1 immunohistochemical expression scoring

**Figure 1:**
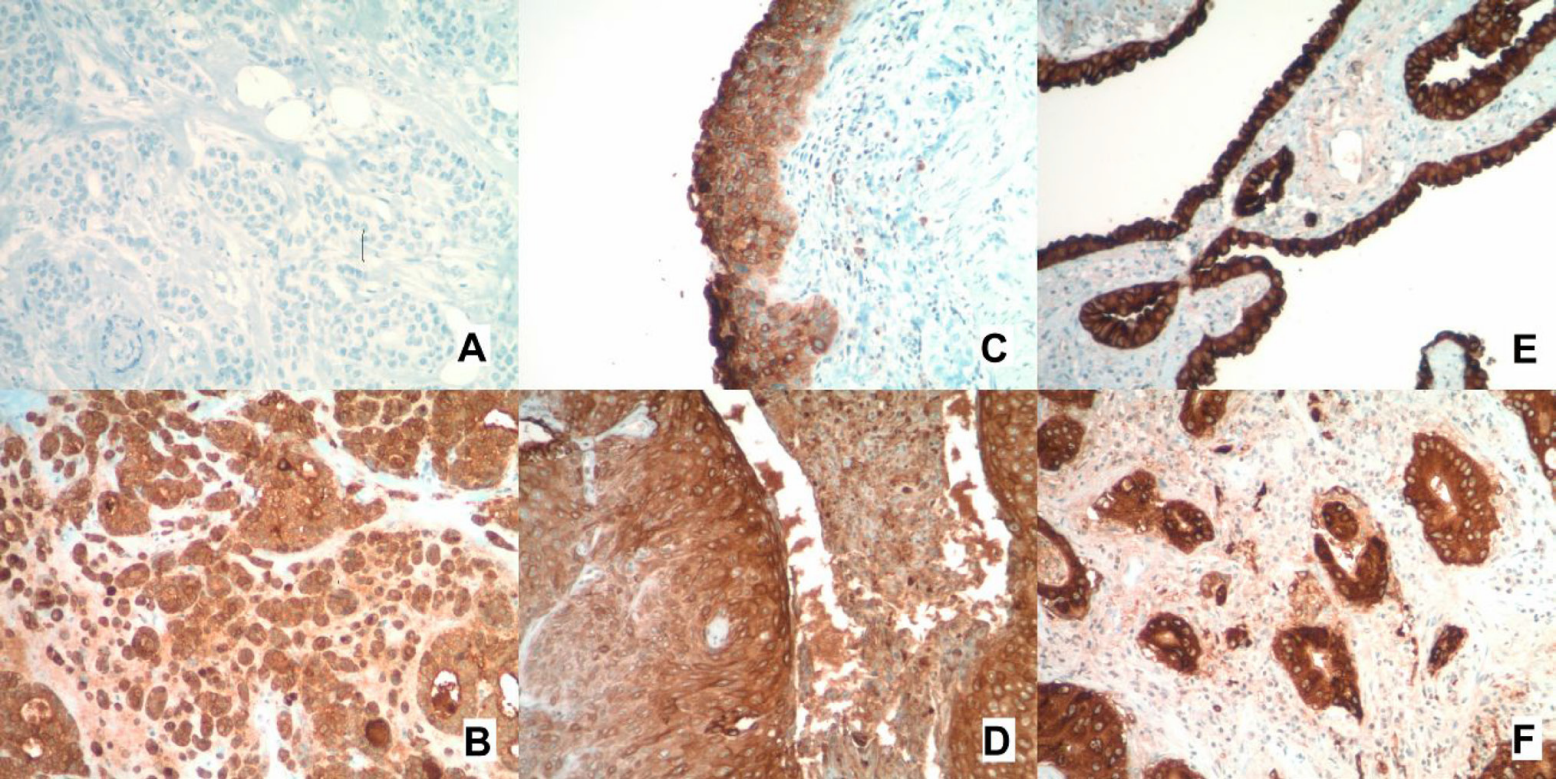
MUC1 immunohistochemistry staining (**A**) Negative control. (**B**) Positive control. (**C**) Positive staining in a region of squamous dysplasia. (**D**) Positive staining in a region of squamous metaplasia. (**E**) Positive staining in a region of adenocarcinoma *in situ*. (**F**) Positive staining in a region of adenocarcinoma.

Figure 1 shows characteristic immunohistochemical (IHC) staining for MUC1 expression. Table 2 summarizes the samples available for paired comparison and the MUC1 IHC expression scores. Among squamous lesions, MUC1 expression scores were significantly increased in dysplastic areas compared with metaplastic areas (mean difference = 0.83, 95% confidence interval [CI], 0.21 to infinity; *P* = 0.021). MUC1 expression levels among areas of squamous cell carcinoma were also increased versus dysplastic areas (mean difference = 0.44, 95% CI, −0.006 to infinity; *P* = 0.052). Among adenocarcinoma lesions, MUC1 expression levels were increased in adenocarcinoma versus adenocarcinoma *in situ* (AIS), although not significantly (mean difference = 0.20, 95% CI, −0.055 to infinity, *P* = 0.094).

**Table 2:** MUC1 immunohistochemistry scoring for primary analysis

Squamous	Carcinoma		Dysplasia		Metaplasia
No. of samples (mean)	15 (2.133)		10 (1.9)		6 (1.333)
*P* value (no. of paired samples)		0.0519 (9)		0.0211 (6)	
**Adenocarcinoma**	**Carcinoma**		**AIS**		
No. of samples (mean)	16 (2.625)		18 (2.389)		
*P* value (no. of paired samples)		0.0944 (15)			

*P* values are for paired *t*-test comparison between overlapping groups. AIS, adenocarcinoma *in situ*.

### Associations between MUC1 expression scores and clinical characteristics

**Figure 2:**
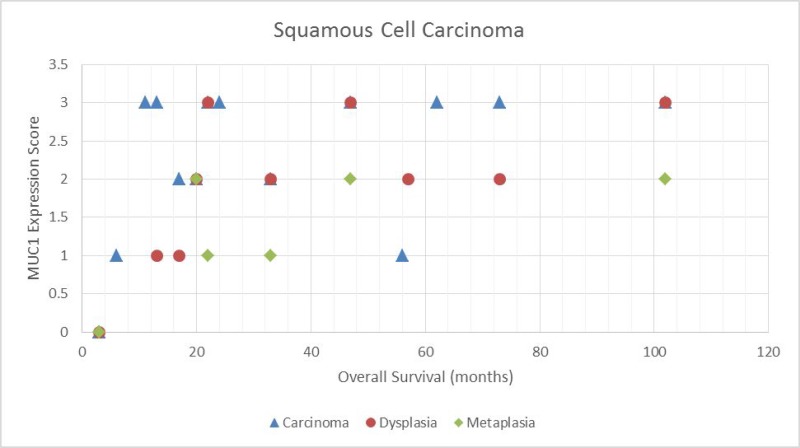
Scatter plot demonstrating the relationship between MUC1 expressions score and overall survival for squamous tumors

**Figure 3:**
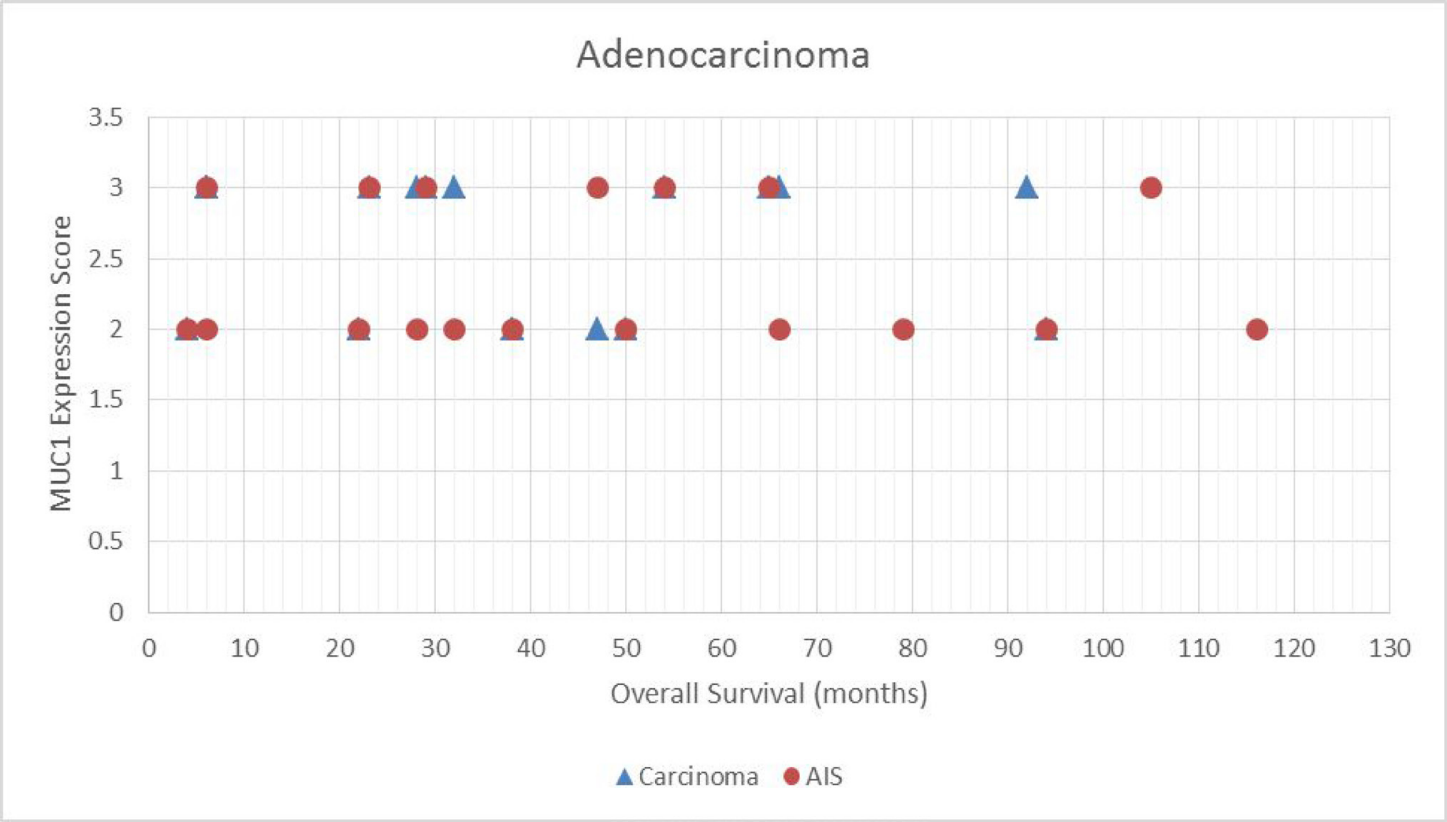
Scatter plot demonstrating the relationship between MUC1 expressions score and overall survival for adenocarcinoma *in situ* (AIS) tumors

Data for up to 116 months of follow-up were available for study patients. Figure 2 shows the association between MUC1 expression score and overall survival for squamous lesions, with corresponding results for adenocarcinoma lesions shown in Figure 3. We observed a statistically significant positive correlation between MUC1 expression and survival in patients with squamous tumors according to the Spearman correlation test (*P* = 0.020 for carcinoma score and *P* = 0.008 for dysplasia score). However, no significant correlation was observed between MUC1 expression and survival in patients with adenocarcinoma (*P* = 0.81).

We used univariate analysis to compare MUC1 expression levels versus age, sex, smoking history, and tumor stage (Table 3). No significant associations were shown between any of these factors and level of MUC1 expression in either squamous or adenocarcinoma tumors.

**Table 3:** Associations between MUC1 expression scores and specific clinical characteristics

	Squamous					
	Carcinoma		Dysplasia		Metaplasia	
Age						
<70 years	2.1 (10)	*p* = 0.878	1.9 (8)	*p* = 0.884	1.4 (5)	^*^
≥70 years	2.2 (5)		2.0 (2)		1.0 (1)	
Sex						
Male	1.9 (8)	*p* = 0.361	1.6 (5)	*p* = 0.371	1.0 (1)	^*^
Female	2.4 (7)		2.2 (5)		1.4 (1)	
Smoking						
<50 pack-years	2.7 (6)	*p* = 0.139	2.2 (6)	*p* = 0.327	1.7 (3)	^*^
≥50 pack years	1.8 (9)		1.5 (4)		1.0 (3)	
Tumor stage						
Stage I	2.2 (9)	*p* = 0.723	2.0 (8)	*p* = 0.557	1.2 (5)	^*^
Stages II–IV	2.0 (6)		1.5 (2)		2.0 (1)	
**Adenocarcinoma**						
	**Carcinoma**		**Adenocarcinoma *in situ***			
Age						
<70 years	2.7 (7)	*p* = 0.547	2.4 (9)	*p* = 0.653		
≥70 years	2.6 (9)		2.3 (9)			
Sex						
Male	2.4 (5)	*p* = 0.237	2.3 (7)	*p* = 0.503		
Female	2.7 (11)		2.5 (11)			
Smoking						
≤25 pack years	2.9 (7)	*p* = 0.103	2.4 (8)	*p* = 0.920		
>25 pack years	2.4 (9)		2.4 (10)			
Tumor stage						
Stage I	2.8 (12)	*p* = 0.082	2.5 (12)	*p* = 0.192		
Stages II–IV	2.3 (4)		2.2 (6)			

Values are expressed as mean immunohistochemistry expression score (with number of samples shown in parentheses).

^*^p values for comparisons of metaplasia in squamous lesions were not reliable since the sample size was too small.

## DISCUSSION

In this analysis of tissue samples from patients with both squamous carcinoma and adenocarcinoma NSCLC, we confirmed that MUC1 was overexpressed in nearby areas of pre-invasive disease and that MUC1 expression was significantly increased in regions of carcinoma compared with adjacent regions of developing premalignant lesions. To our knowledge, this is the first report to specifically analyze and demonstrate increased MUC1 expression alongside increasing carcinogenesis of pulmonary lesions in human lung cancer.

An interesting finding that we observed in the present study was the correlation between squamous carcinoma and dysplasia MUC1 expression and increased overall survival. This finding is in contrast to other literature as discussed above [14, 18–20]. One patient in the present study with confirmed squamous cell carcinoma and strong (3+) MUC1 expression exhibited a prolonged, durable response to treatment; this treatment currently involves therapy with a PD-1 inhibitor. There were 13 patients in the present study who were lost to follow-up, and it is not known whether any of these patients may have been also treated with immunotherapy. Previous studies observing the negative association between MUC1 expression and survival in lung cancer were conducted before the recent use of checkpoint inhibitor therapy [18–20, 22]. It seems plausible that increased aberrant MUC1 cell surface expression may render a tumor more immunogenic; however, whether this increased expression actually translates into improved response to immunotherapeutic approaches remains unknown.

As discussed above, the association between MUC1 overexpression and tumorigenesis of lung and other cancers has been demonstrated in hamster and mouse models [10, 17], and these findings have been extrapolated to human lung cancer. MUC1 has previously been described as aberrantly expressed in premalignant lung lesions in squamous and adenocarcinoma NSCLC [23], and differential depolarized expression of MUC1 has been observed between primary lung tumors and metastatic lesions [24]. In a study that analyzed the gene expression of mucins, including MUC1, in squamous NSCLC, pre-invasive squamous lesions (primarily from separate clinical samples), and normal respiratory epithelium, MUC1 expression was weak in pre-invasive and invasive lesions, with no significant increase shown in invasive carcinoma. However, the study characterized MUC1 expression with mRNA *in situ* hybridization rather than with IHC [25]. In another study that investigated MUC1 expression by IHC in squamous adenocarcinomas of the lung and in adjacent pre-invasive lesions, MUC1 was highly expressed in most pre-invasive lesions, and expression levels in the invasive components were strongly associated with the presence of pre-invasive lesions. However, no quantitative comparisons were conducted of the differential expression between pre-invasive and invasive lesions [26].

Our demonstration of increased MUC1 expression in invasive and premalignant lesions in paired samples in human NSCLC, along with progression of tumorigenesis, adds to the body of research supporting the role of MUC1 in the development of human lung cancer. This possible role has spurred the investigation of vaccines directed against MUC1 as an immunomodulatory approach to lung cancer treatment and prevention. The use of MUC1 peptide vaccines in human cancer patients has been demonstrated to generate both anti-MUC1 antibody and cytotoxic T-lymphocyte responses [27].

Several vaccines utilizing MUC1 as an antigen have been developed and investigated to date. TG4010 (a modified vaccinia virus expressing MUC1 and interleukin 2) was studied in a phase 2 trial in combination with first-line chemotherapy in patients with stage IV untreated NSCLC. This trial demonstrated a small but statistically significant improvement in progression-free survival from 5.1 to 5.9 months (*P* = 0.019) [28, 29]. Tecemotide (L-BLP25, a vaccine utilizing the MUC1-derived 25-amino acid L-nBLP25) was investigated in the START trial, in which patients with unresectable stage III NSCLC were randomized to vaccination versus placebo after treatment with platinum-based chemotherapy and radiation. Results from the trial showed an improved overall survival from 22.3 to 25.6 months, although not significant (*P* = 0.123). However, in a preplanned subgroup analysis of those patients who received concurrent (rather than sequential) chemoradiotherapy, overall survival increased significantly from 20.6 to 30.8 months (*P* = 0.016) [30, 31].

Vaccine strategies with targets other than MUC1 have been used for the treatment of advanced-stage NSCLC, with similar overall results, showing at best a modest benefit or a benefit for only a subgroup of patients [32–36]. Although clinical success with a vaccination approach has been limited, interest in MUC1 as the target for immune therapy continues, given the evidence that certain subgroups of patients may exhibit meaningful responses. Two clinical trials investigating the combination of these vaccinations with the checkpoint inhibitor nivolumab in advanced NSCLC are ongoing: one with TG4010 (https://clinicaltrials.gov/ NCT02823990) and one with CV301 (NCT02840994), a vaccine targeting both MUC1 and carcinoembryonic antigen.

Despite past negative trials investigating chemoprevention with beta-carotene or its derivatives for NSCLC [37, 38], interest in chemoprevention continues, with multiple trials still ongoing [39]. Vaccination as a “chemoprevention” strategy in high-risk populations may prove to be a viable strategy, particularly if antigenic expression targeted by a vaccine is shown to be expressed in premalignant lesions as a part of the tumorigenesis of lung cancers. Furthermore, the low toxicity profile of vaccinations is appealing. Lung cancer chemoprevention trials follow the model of multistep carcinogenesis as defined for invasive squamous carcinoma: a progression from normal, to hyperplasia, to metaplasia, to increasing degrees of dysplasia, to carcinoma *in situ*, and finally to invasive carcinoma [39]. The observation that MUC1 overexpression begins in metaplastic and dysplastic lesions and in carcinoma *in situ* suggests that a MUC1 peptide-based vaccination may be useful as a chemoprevention strategy. Although such an approach has not yet been investigated in lung cancer, there has been one study that reported use of a MUC1-based vaccine in patients at high risk for adenocarcinoma of the colon [40]. Follow-up from this colon cancer study demonstrated that this vaccination can generate a specific and durable immune response, although it remains to be seen whether this will translate into a clinical benefit.

Our study has several limitations. Although our study was powered to detect an observable significant difference in MUC1 expression between malignant and premalignant lesions and although clinical characteristics and outcomes were collected for correlation with MUC1 expression scores, the sample size was limited. Three patient samples were excluded from analysis due to miscategorized histology; in addition, several patients were lost to long-term follow-up. It is important to note that, given the observational nature of this study and its small sample size, data regarding clinical associations of MUC1 expression and correlation with survival should be regarded only as hypothesis-generating at this point.

The use of an IHC score (derived as the product of the intensity of staining and the percentage of stained cells) was contemplated for quantification of MUC1 expression scoring [41]. However, because the areas of varying differentiation (metaplasia, dysplasia, and carcinoma) were often limited, quantification of the staining percentage within each component was deemed to be highly inaccurate. The MUC1 antibody used in the present study has specificity for MUC1-N. In light of the recently published evidence of MUC1-C's role in cellular signaling, and the fact that MUC1-N may be shed into circulation and MUC1-C remains membrane-bound [12, 13], replication of these results with an antibody against MUC1-C could be an important area of future study.

MUC1 overexpression appears to be increased with the progression of premalignant lung lesions to invasive carcinoma in patients with NSCLC (significantly for squamous cell carcinoma and with a trend toward significance for adenocarcinoma). This is consistent with the rationale for MUC1 as a potential therapeutic target for novel efforts to suppress or prevent the development of lung cancer. We anticipate multiple avenues for future research of clinical therapies targeting MUC1 in lung cancer.

## MATERIALS AND METHODS

### Tissue sources

Tissue was obtained from the Moffitt Cancer Center Tissue Bank or the Moffitt Anatomic Pathology Department when necessary. Tissue samples from both sources came from patients who had provided written informed consent to the Moffitt Cancer Center Total Cancer Care protocol or the Moffitt general tumor banking protocol. Thus, all tissue samples were obtained from patients who underwent a surgical or interventional procedure outside the context of this study, which in nearly all cases was a resection for curative intent in patients with early-stage clinical disease.

Tumor tissue and adjacent non-tumor tissue samples were formalin-fixed/paraffin-embedded or snap frozen. Tissue blocks of both normal tissue and tumor tissue were requested from each patient undergoing a surgical procedure or biopsy for all stages of NSCLC (stages I to IV); when feasible, adjacent metaplastic, dysplastic, and AIS areas were also obtained. The unstained sections were cut at traditional 3-μm-thick slices. All assays were performed in the Moffitt Cancer Center.

### Tissue marker studies

MUC1 expression was assessed by IHC. The anti-MUC1 antibody 4H5 (with specificity against abnormal hypoglycosylated MUC1-N terminal subunit) was used for the purposes of IHC, as previously described [42]. The IHC staining procedures were performed manually at room temperature using avidin-biotin-peroxidase complex methods (Vectastatin Elite ABC kit; Vector Lab, Burlingame, CA). After sample slides were washed with phosphate-buffered saline for 5 minutes, slides were blocked with normal serum with 3% bovine serum albumin for 10 minutes followed by incubation with the primary antibody, rinsed, and incubated with a biotinylated secondary antibody and washed again. Slides were incubated with the avidin-biotin complex for 1 hour and washed again. Chromogen was developed with 3,3-diaminobenzidine (DAB) (DAB substrate kit for peroxidase; Vector Lab). Automated quantitative analysis was used as a quantitative imaging analysis tool for marker expression [43, 44]. Levels of MUC1 expression in areas of dysplasia, metaplasia, AIS, and carcinoma present within the same tissue sample were characterized independently. MUC1 expression levels were quantitated numerically with a score of 0, 1, 2, and 3, corresponding to negative, weak, moderate, and strong staining intensity, respectively.

### Clinical data

We obtained patient demographic and treatment data from medical records, including age, sex, performance status at presentation, tumor stage before and after surgical resection, histology (including adenocarcinoma, squamous cell carcinoma, large cell carcinoma, or other; metaplasia, dysplasia, and/or AIS), grade, number and type of therapies (chemotherapy/radiation/surgery), response to chemotherapy, disease-free interval, and overall survival. All data were collected under an Institutional Review Board-approved protocol.

### Statistical analyses

A plan for analysis of 40 tumor samples was made (20 squamous tumors and 20 adenocarcinoma tumors), anticipating that there would be presence of both premalignant and malignant regions within the same sample for at least 16 samples for each tumor type. For alpha = 0.05, this would provide 80% power to detect a 0.65 standard deviation or greater increase in the dysplastic mean compared with the metaplastic mean.

For the primary analysis, MUC1 expression scores for paired samples (areas of differing histology within the same patient) were compared using the paired sample *t* test. Secondary analyses included determining correlations between MUC1 expression scores and overall survival, which were quantified using the Spearman rank correlation coefficient. In addition, the *t* test was applied to assess for correlations between MUC1 expression scores and patient characteristics, including smoking history, age, sex, and tumor stage. Clinical data as mentioned above, including patient characteristics, staging, treatment, and survival, were characterized using descriptive statistics.

## Abbreviations

AIS: Adenocarcinoma *in situ*
CI: Confidence interval
DAB: 3,3-Diaminobenzidine
IHC: Immunohistochemistry
MUC1: Mucin 1
MUC1-C: Mucin 1 C-terminal subunit
MUC1-N: Mucin 1 N-terminal subunit
NSCLC: Non-small cell lung cancer
